# Biotransformation of Antibiotics by *Coriolopsis gallica*: Degradation of Compounds Does Not Always Eliminate Their Toxicity

**DOI:** 10.3390/antibiotics14090897

**Published:** 2025-09-05

**Authors:** Bouthaina Ghariani, Héla Zouari-Mechichi, Abdulrahman H. Alessa, Hussain Alqahtani, Ahmad A. Alsaigh, Tahar Mechichi

**Affiliations:** 1 Laboratory of Biochemistry and Enzyme Engineering of Lipases, National School of Engineers of Sfax, BP 1173, University of Sfax, Sfax 3038, Tunisia; hela.zouari@isbs.usf.tn; 2 Institute of Biotechnology of Sfax, BP “1175”, University of Sfax, Sfax 3038, Tunisia; 3 Department of Biology, Faculty of Science, University of Tabuk, Tabuk 71491, Saudi Arabia; alessiabdulrahman@gmail.com (A.H.A.); h.alqahtani@ut.edu.sa (H.A.); 4 Department of Biology, Faculty of Science, Umm Al-Qura University, Makkah 24381, Saudi Arabia; aassaigh@uqu.edu.sa

**Keywords:** antibiotics, *Coriolopsis gallica*, biotransformation, detoxification, Laccase

## Abstract

Background/Objectives: Wastewaters containing antibiotics pose risks to human health and soil ecosystems. In this study, the white-rot fungus *Coriolopsis gallica* (a basidiomycete exhibiting high laccase production) was used for the biotransformation of three antibiotics (50 mg L^−1^): tetracycline, chloramphenicol, and sulfanilamide. Methods: The biotransformation process was investigated in liquid and solid media using high-performance liquid chromatography (HPLC) and the bacterial growth inhibition agar well diffusion method, respectively. Results: Among the three antibiotics tested, tetracycline showed the highest biotransformation efficiency, achieving a 100% removal rate in the liquid medium and a 100% decrease in the growth inhibition of *Escherichia coli* in the solid medium. Chloramphenicol and sulfanilamide were partially removed (20% and 16%, respectively) after 12 days of treatment in more than one step without the loss of their antibacterial activities. The presence of these antibiotics in the culture medium of *C. gallica* enhanced laccase activity, indicating that this ligninolytic enzyme might participate in the biotransformation process. Conclusions: Thus, the results reported in this article extend our knowledge of the catalytic potential of *C. gallica* and give further perspectives for its application in the biodegradation of antibiotics. To the best of our knowledge, this is the first study wherein *C. gallica* was used for the treatment of tetracycline, chloramphenicol, and sulfanilamide.

## 1. Introduction

The use of pharmaceutical compounds for human and veterinary purposes, especially antibiotics, has continuously increased in recent years [1]. In fact, the World Health Organization has predicted a worldwide increase in antibiotic consumption from 63.2 to 105.3 thousand tons by 2030 [2]. This increase has been reported to be the result of an incremental increase in the unnecessary use of antibiotics [3]. As a result, the concentrations of these compounds in aquatic ecosystems, such as municipal wastewater treatment systems, surface water, and groundwater, have also increased [4,5].

Antibiotics make their way into aquatic systems through different sources, such as wastewater treatment plants, antibiotic manufacturing plants, hospitals, and livestock farms, with the potential to exert selection pressure on bacteria and enhance their antimicrobial resistance [6,7]. Several studies have shown that wastewater from municipal conventional wastewater treatment plants (WWTPs) constitutes a significant source of contamination of the aquatic environment by antibacterial agents because WWTPs are not designed to remove antibacterial agents from wastewater [8]. Therefore, antibiotics have been detected in different environmental matrices, such as groundwater and surface water [9], soil and sediment samples [10], and aquatic organisms [11]. They show pseudo-persistent behavior in the environment, and their accumulation in different environments can threaten human and animal existence and may have an impact on health [12]. Indeed, using treated wastewater for irrigation may lead to the contamination of agricultural soils and an uptake of residual antibiotics by plants [13]. As a result, antibiotic residues present in the ecosystem provide an ideal setting for the acquisition and spread of antibiotic resistance genes, posing serious environmental problems [14].

Based on data from 87 countries that was reported in 2020, a new report from the World Health Organization noted high levels of resistance in bacteria, leading to life-threatening blood infections, as well as an increase in the treatment resistance of several bacteria responsible for common infections in the population [15]. Accordingly, the persistence of antibiotics, especially in the aquatic matrix, should provoke world leaders and the scientific community to take necessary actions and develop technologies for remedying these issues in order to protect the environment [16].

Tetracycline, chloramphenicol, and sulfanilamide, which are used in this study, are widely used inhuman and veterinary medicine. Tetracycline is widely used in human medicine, with a high annual global consumption figure [17]. It is resistant to biological treatments with elimination rates of 80–90 [18], and its abiotic degradation under natural conditions is very slow. Chloramphenicol is a broad-spectrum antibiotic that is extensively administered in veterinary medicine [19]. It is characterized by its incomplete metabolism by human beings and animals; hence, unmetabolized chloramphenicol (30–90%) is excreted into the environment through urine and excrement [20]. As a recalcitrant antibiotic with a stable structure, it survives WWTP treatment and has been detected, along with its metabolites, in different environmental matrices and in foods [6]. Thus, it is necessary to seek a highly efficient strategy for reducing or eliminating chloramphenicol residues in the environment. Like all sulfonamides, sulfanilamide, a breakdown product of other sulfonamides and dyes, has been detected in all aquatic systems [21]. At the Sfax WWTP, sulfanilamide was not detected; however, sulfapyridine and sulfamethoxazole were detected at concentrations greater than 365.50 and 126.50 ng L^−1^, respectively. After treatment, these concentrations were reduced, with elimination rates of around 61% and 33%, respectively [2].

Many studies published in recent years have focused on the removal of antibiotics via chemical processes such as advanced oxidation processes [22,23,24], Fenton and Fenton-like reactions [25,26], ozonation [27], electro-chemical processes [28,29], photo-degradation [30,31,32], sonochemical degradation, chlorination, and physical processes such as adsorption and filtration [33] or the combination of the two [34]. In spite of these methods’ moderate costs and simplicity, the degradation of the target antibiotics usually remains incomplete [35]; they also generate toxic products [36], and, in the case of the adsorption process, there is simply a transfer of the pollutant from an aqueous phase to a solid one. To prevent these disadvantages, microbial organisms can be applied as an effective alternative for degrading antibiotics and attenuating their toxicity [37]. Bioremediation is considered the most eco-friendly and low-cost approach to removing pollutants [38] such as textile dyes [39], pesticides [40], microplastics [41], heavy metals [42], and pharmaceuticals [43], especially antibiotics [44], using processes involving natural mechanisms. Different microorganisms have been used to remove antibiotics, such as bacteria [45], yeast [46], algae [47], and fungi [48]. Bioremediation techniques form part of the triple bottom line, providing economic, environmental, and social benefits [43].

White-rot fungi (WRF) are a promising group of fungi known for their ability to transform recalcitrant compounds with aromatic structures [49], such as textile dye derivatives. These organisms have the potential to adsorb, transform, and mineralize a large spectrum of antibiotics [50,51] via non-specific enzymes such as laccase, peroxidases [52] and cytochrome P450 system [53]. This oxidizing property could have useful applications in the removal of antibiotics and seems to be a very promising approach to improving water effluent quality at WWTPs.

The objective of this work was to investigate the ability of *Coriolopsis gallica,* a fungal strain known for its considerable laccase production and previously applied for the biotransformation of recalcitrant antibiotics [54,55], to transform and inactivate three representative antibiotics belonging to different drug classes, namely, tetracyclines, phenicols, and sulfonamides, which share an aromatic structure.

## 2. Results

### 2.1. Antibiotic Biotransformation by C. gallica

**Figure 1:**
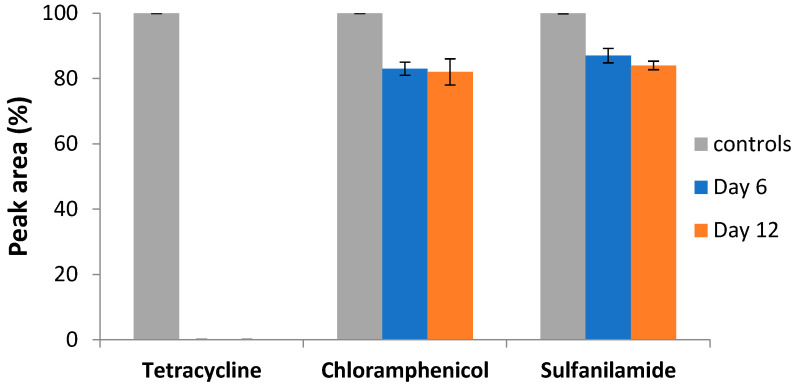
Antibiotic degradation by *Coriolopsis gallica* after 6 days (Day 6) and 12 days (Day 12) of treatment; controls: culture media (M7) supplemented with 50 mg L^−1^ of antibiotics. Each data point (mean ± standard deviation) is the result of triplicate experiments.

In this study, we investigated the potential of *C. gallica* to biotransform three types of antibiotics over a 12-day incubation period. Residual concentrations of tetracycline, chloramphenicol, and sulfanilamide were estimated by conducting an HPLC-UV-MS analysis (270 nm) on supernatants after 6 and 12 days of incubation at 30 °C (Figure 1).

Untreated antibiotics (controls) showed no degradation over 12 days of incubation at 30 °C (Figure 1). There was a slight decrease in the peak areas of chloramphenicol (20%) and sulfanilamide (16%) compared to their controls after the same period of incubation. However, tetracycline was effectively removed (100%) after 6 days of treatment.

### 2.2. Detection of Degradation Metabolites

**Figure 2:**
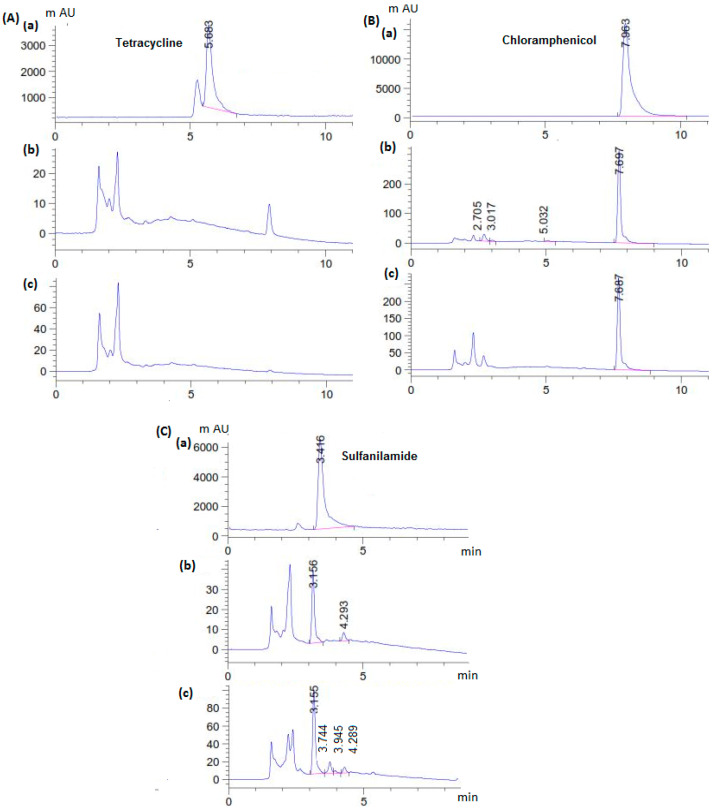
Time-course chromatograms corresponding to (**A**) tetracycline, (**B**) chloramphenicol, and (**C**) sulfanilamide treated by *C. gallica*; (**a**) Control, after (**b**) 6 days and (**c**) 12 days of treatment. Culture conditions were as follows: 50 mg L^−1^ of antibiotics, M7, 0.15 mM Cu^2+^, 30 °C, and 150 rpm.

*C. gallica* secretomes from culture media supplemented with antibiotics on days 6 and 12 after treatment were studied via HPLC-UV-MS to detect the biotransformation metabolites. The chromatograms obtained are shown in Figure 2.

Time-course chromatograms corresponding to the culture medium supplemented with antibiotics showed that they had been transformed by *C. gallica* over the 12 days of treatment. The antibiotic biotransformation profiles varied significantly depending on the structure of the antibiotic. The appearance of low-molecular weight molecular fragments from parent antibiotics in liquid chromatography–mass spectrometry confirmed the biodegradation process. Tetracycline (5.6 min) had been completely removed (100%) after 6 days of treatment, with the appearance of a new by-product eluted at 1.5 min (Figure 2A). For chloramphenicol (Figure 2B) and sulfanilamide (Figure 2C), characteristic peaks of each antibiotic were still detected on UV-chromatograms on days 6 and 12 after treatment, with the appearance of new ones, corresponding to their biodegradation metabolites. The chromatograms obtained on day 6 were different from those recorded on day 12 after treatment, suggesting that the transformation of antibiotics occurred in several stages.

### 2.3. Laccase-like Activity in Submerged Fugal Cultures

**Figure 3:**
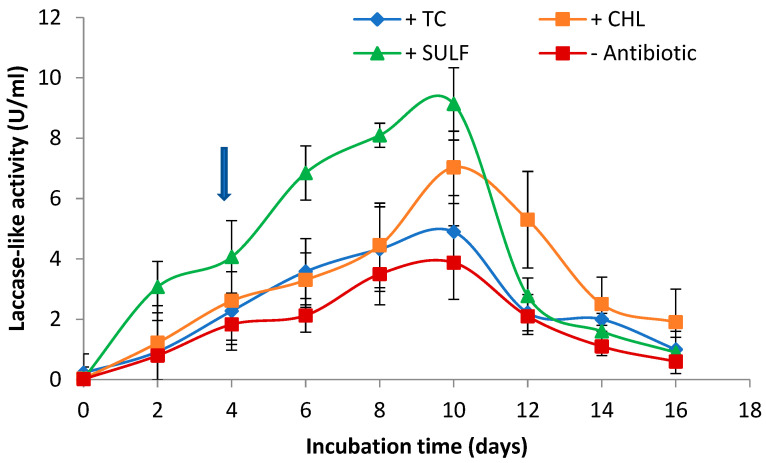
Kinetics of laccase-like activity under both conditions: with or without antibiotics (TC, CHL, and SULF). TC: tetracycline, CHL: chloramphenicol; SULF: sulfanilamide. 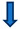: Antibiotics addition in the culture medium. Each data point (mean ± standard deviation) is the result of triplicate experiments.

Laccase is an oxidative enzyme that is produced by *C. gallica* and may be involved in antibiotic degradation. Laccase-like activity was measured over 12 days of treatment in submerged cultures under both the conditions tested (with or without antibiotics), as illustrated in Figure 3.

For all conditions, laccase-like activity increased during the fungal culture until reaching a maximum by around day 10, with significant differences found between the controls (3.88 U mL^−1^) and the cultures supplemented with antibiotics (4.9 U mL^−1^, 7 U mL^−1^, and 9.14 U mL^−1^ for TC, CHL, and SULF, respectively). The presence of antibiotics in the culture media of *C. gallica* induced laccase production. This finding suggests that laccase could be involved in the biodegradation of tetracycline, chloramphenicol, and sulfanilamide.

### 2.4. In Vitro Analysis of Residual Antibiotics

**Figure 4:**
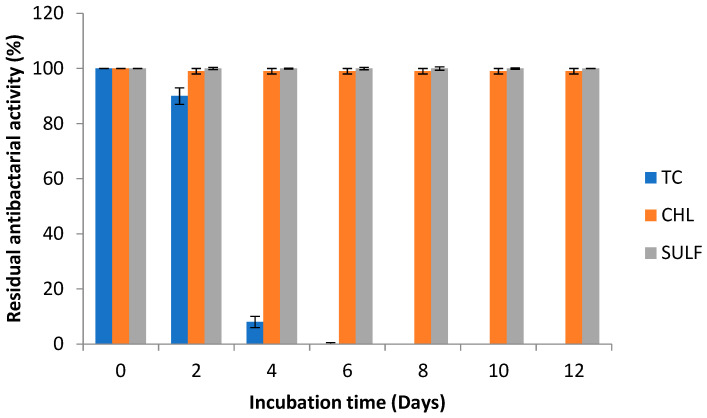
Kinetics of antibacterial activity removal in *C. gallica* cultures over 12 days of treatment (Day 0 is the day on where *C. gallica* cultures were supplemented with antibiotics). Data are means ± standard deviations of triplicate experiments.

As the biotransformation of an antibiotic does not necessarily translate into a reduction in its antimicrobial activity, we evaluated the antibacterial activities of the antibiotics on a solid *Escherichia coli-* spread medium loaded with a 50 µL aliquot of the culture medium containing antibiotics at 50 mg L^−1^ during treatment with *C. gallica* cultures. The *E. coli* growth inhibition zones related to antibiotics treated with the fungus were measured, and the results are reported in Figure 4.

The results showed that the best removal yield was obtained for tetracycline, with a 100% reduction in the growth inhibition zone against *E. coli* after 6 days of treatment. The residual antibiotic activity of tetracycline seemed to be proportional to its concentration in the culture medium. The intermediates generated since day 6 did not inhibit the growth of *E. coli*. However, both chloramphenicol and sulfanilamide resulted in the same degree of inhibition-zone reduction as the controls during culture incubation.

## 3. Discussion

White-rot fungi were investigated for their use in bioremediation because of their ability to degrade not only lignocellulosic compounds but also other pollutants, such as aromatic compounds, munitions, and azide. The aim of this study was to evaluate the potential of the white-rot fungus *C. gallica* to bio-transform three representative frequently used antibiotics. This strain has already been tested for its ability to remove fluoroquinolones [54] and ß-lactams [55].

In this study, *C. gallica* achieved complete tetracycline biotransformation in the solid and liquid media after 6 days of treatment. However, chloramphenicol and sulfanilamide were only partially transformed, and their antibacterial activities remained intact. Several studies have investigated the biotransformation of tetracyclines and sulfonamides by white-rot fungi. In comparison with other species of WRF, *Phaerochaetechrysosporium* was able to transform only 31% of tetracycline (100 mgL^−1^) after 14 days of treatment in a liquid medium [56]. Also, *Bjerkanderaadusta* was found to significantly degrade tetracycline (up to 92%) [57]. Recently *Aspergillussp*. LS-1 has been found to be able to degrade chlortetracycline 95.41% within 72 h [58]. *Pleurotuseryngii* needed 7 days to reduce > 90% of three sulfonamides [59]. Meanwhile, *Tramets versicolor* biotransforms sulfacetamide (300 mg L^−1^) after 7 days of treatment [60]. However, there is only scant information available about the biodegradation of chloramphenicol by fungi, especially by white-rot fungi, and most microbial biodegradation methods focus on bacterial biodegradation. This recalcitrant antibiotic has been partially bio-transformed by the endophytic strains *Aspergillussp*. and *Trichodermasp*. (29.3% and 25.2%, respectively) after 9 days of incubation [61], and a recent study reported that *T. versicolor* was able to remove 44% of chloramphenicol (5 mg L^−1^) after 15 days of treatment via degradation and adsorption processes [62].

In previous studies, *C. gallica* was found to be able to completely remove ampicillin [55] and partially remove levofloxacin under the same conditions (M7, 50 mg L^−1^) via an oxidative process [54]. This strain’s ability to reduce different antibiotics’ structures highlights its potential for use as an effective antibiotic attacker. These findings converge with previous studies reporting that this fungal species has the potential to degrade phenolic pollutants such as industrial textile dyes, olive meal wastewater, Bisphenol A and diesel fuels [63,64,65,66]. *C. gallica* showed promising results, significantly transforming ampicillin and tetracycline molecules. However, it faced challenges in efficiently removing chloramphenicol and sulfanilamide. This result can be explained by the recalcitrant nature of these antibiotics [62,67]. Also, functional groups may contribute to the electronic effects that hinder degradation by affecting the interaction between contaminant molecules and enzymes. With an increase in the electronegativity of these molecules, degradation rates decrease [68,69]. This finding emphasizes the importance of evaluating the degradation potential of such fungal strains for specific antibiotics.

Antibiotic removal via fungal treatment can be carried out using different processes, such as adsorption, mineralization, and biotransformation/biodegradation mediated by enzymatic systems [70]. In this study, we focused on the biotransformation of tetracycline, chloramphenicol, and sulfanilamide by extracellular enzymes involved in lignin degradation, essentially laccases, as no peroxidase-like activity was detected in the *C. gallica* culture [54]. We found that the presence of these antibiotics enhanced laccase-like activity relative to the control, suggesting that this enzyme could be involved in antibiotic biodegradation. A similar result was obtained in the treatment of ampicillin with a *C. gallica* culture under the same conditions [55]. These findings are in agreement with those of previous studies in which a proteomic analysis suggested that extracellular enzymes could be involved in antibiotic degradation, with a putative major role identified for laccases [54]. Nevertheless, other enzymes could be involved in antibiotic degradation, such as the peroxidases MnP and LiP [71], members of the cytochrome P450 system [72], and enzymes responsible for mycelial adsorption [48]. Laccase has previously been used in the biotransformation of antibiotics. This enzyme can easily be repurposed as a free or immobilized system to support sustainable processes [73]. Many studies have used laccase to degrade antibiotics like tetracyclines [73,74,75], phenicols [76], and sulfonamides [77], supporting our suggestion that laccase from *C. gallica* might be the key enzyme in the biotransformation of these antibiotics. Therefore, it would be necessary to carry out an in vitro experiment using purified laccase in order to validate this suggestion.

Since the biotransformation of antibiotics does not always mean detoxification, the residual antibacterial activity of the treated solutions was investigated to ensure the efficiency of the treatment. In this research, we focused on this activity, as antibiotic-resistant bacteria and genes have become a major threat to human health worldwide [78]. Tetracycline activity was effectively removed by the *C. gallica* culture after 6 days of treatment. This result suggests that biotransformation may have occurred on functional groups that are essential for tetracycline’s mechanism of action, namely, the ß-diketone/enol chelating system spanning C-1, C-3, and C-11-12a through oxidation or the dimethylamino group at C-4 transformation [69,79]. This suggestion is supported by the findings of previous studies that investigated the detoxification of tetracyclines using fungal cultures, their enzymes, or crude mycelial culture [73,74,75,80]. These studies found that tetracyclines were degraded into low-toxicity or non-toxic small-molecular products through a series of reactions such as demethylation, deamination, dehydration, hydroxylation, oxidation, and ring opening [74]. However, chloramphenicol and sulfanilamide retained their initial antibacterial activity despite the reduced concentrations, indicating that biotransformation may have occurred at non-functional groups required for activity, preserving the nitro group and dichloroacetamide moiety in the case of chloramphenicol and the sulfonamide group –SO_2_NH_2_ and the aromatic amine –NH_2_ in the case of sulfanilamide. In addition, residual parent molecules may still be present at levels above the minimum inhibitory concentration for *E. coli* or biotransformation-generated active metabolites. A recent study reported that the biodegradation of chloramphenicol by *Trichoderma sp*. generated the main metabolite 4-nitrobenzaldehyde, which presented higher ecotoxicity to green algae than the initial antibiotic [61]. However, the biotransformation of chloramphenicol by laccase from *Trameteshirsuta* generated aldehyde in a form non-toxic to micro-organisms [76].

Ligninolytic fungi are known for their ability to remove several antibiotics and diminish their antibacterial activities. Despite the importance of the results obtained, this study has several significant limitations that must be considered when interpreting the results. First, further experiments with purified laccase should be performed to confirm the role of this enzyme in the biotransformation of tetracycline, chloramphenicol, and sulfanilamide antibiotics. Second, previous studies showed that other oxidative enzymes of *C. gallica* contribute, among other mechanisms, to the negation of antibacterial activity. So, further experiments, such as proteomic analyses, should be conducted to investigate enzymes potentially involved in the biotransformation process. Lastly, the loss of antibacterial activity could be attributed to the cleavage of the aromatic ring under the action of laccase. It would be prudent to identify the transformation products generated during treatment with *C. gallica*, determine the biotransformation pathway, and perform ecotoxicity and cytotoxicity tests to confirm detoxification.

## 4. Materials and Methods

### 4.1. Fungal Strain and Culture Media

In this study, the ability of the white-rot fungi *Coriolopsis gallica* CLBE55(ON340792)—a Basidiomycete isolated from a forest biotope in northwestern Tunisia [54,66]—to degrade tetracycline, chloramphenicol, and sulfanilamide was tested. The strain was maintained by sub-culturing it monthly on 2% malt extract agar (MEA) slants at pH 5 and 30 °C (Sigma-Aldrich, St. Louis, MO, USA). The liquid medium used was M7 medium at pH 5.5, as described in [55]. This basal medium contained (per L) glucose (100 g), soya peptone (5 g), yeast extract (1 g), ammonium citrate (2 g), MgSO_4_ (0.5 g), K_2_HPO_4_ (1 g), KCl (0.5 g), and trace element solution (1 mL). The composition of the trace element solution was (g L^−1^) as follows: B_4_O_7_Na_2_ 10H_2_O (0.1), CuSO_4_ 5H_2_O (0.01), FeSO_4_ 7H_2_O (0.05), MnSO_4_ 4H_2_O (0.01), ZnSO_7_ H_2_O (0.07), and MoNH_4_ 4H_2_O (0.01).

The precultures were performed in 25 mL of M7 and inoculated with three agar plugs (6 mm diameter) cut from the growing edge of a plate stock culture and incubated at 30 °C for three days at 150 rpm. The cultures were performed in 500 mL Erlenmeyer flasks containing 150 mL of M7 medium, which was inoculated with the mycelial mixture obtained from the three-day preculture and partially ground down using autoclaved glass beads (0.6 mm). After three days of incubation at 30 °C and 150 rpm, 150 µM CuSO_4_ was added as a laccase inducer. On day 4 of incubation, tetracycline, chloramphenicol, and sulfanilamide were added separately into the fungal cultures at final concentrations of 50 mg L^−1^. All media were sterilized by autoclaving them at 120 °C for 20 min. Each experiment was conducted in triplicate and included non-inoculated controls containing 150 mL of the same medium incubated in the dark(to exclude the influence of light on the antibiotics’ stability) on an orbital checker under the same conditions mentioned earlier. During antibiotic treatment, 2 mL samples were periodically withdrawn, filtered (0.45 µm), and used to assess laccase and antibacterial activity. To follow the residual antibiotics during cultivation, we withdrew the samples on day 6 and day 12 and kept them at −20 °C until the high-performance liquid chromatography (HPLC) analysis was conducted. Liquid Lysogeny broth (LB) medium was used to cultivate *E. coli* cells, and solid LB was used to assay the residual antibacterial activity according to the method described in [55].

### 4.2. Chemicals and Reagents

Tetracycline (CAS No 60-54-8, ≥98.0–102%), chloramphenicol (CAS No 56-75-7, ≥98%), sulfanilamide (CAS No 63-74-1, ≥98%), and 2,6-dimethoxyphenol (2,6 -DMP, 99%) were obtained from Sigma-Aldrich. All other chemicals and solvents used in this study were HPLC-grade. The chemical structures and some characteristics of the studied antibiotics are shown in Table 1.

### 4.3. Evolution of Antibiotic Concentration in the C. gallica Culture Filtrate

The time-course changes in tetracycline, chloramphenicol, and sulfanilamide concentrations were monitored using HPLC-UV-MS in fungal cultures at two different time points (days 6 and 12 of culture). Filtered aliquots of the culture supernatants were injected into an HPLC system (HPLC; Agilent 1100 Series, Agilent Technologies, Santa Clara, CA, USA) coupled with an electrospray ionization mass spectrometer (ESI–MS; Agilent Technologies 61120 Quadrupole LC/MS). Separation was performed using a ZORBAX SB-C18 (150 mm × 4.6 mm, 5 µm) column at 35 °C, as described in [55]. ESI–MS spectra were acquired using an ISQ-EM mass spectrometer (Thermo Scientific, Waltham, Massachusetts, MA, USA), with the vaporizer temperature set to 150 °C, the ion transfer tube temperature set to 300 °C, and the sheath gas pressure set to 60 psig. The antibiotic peaks were assigned based on the retention time of each standard at a wavelength of 270 nm and the mass of the protonated ions. During the treatment of the different antibiotics, two parameters were monitored: laccase like-activity and antibacterial activity removal.

### 4.4. Laccase-like Activity Assay

During the 12 days of treatment, samples of the fungal culture supernatants (with or without antibiotics) were withdrawn every 2 days and centrifuged at 12,000× *g* for 10 min to assay the laccase-like activity, as described in [55]. One unit of enzyme activity was defined as the amount of enzyme oxidizing 1 µM of substrate per minute.

The laccase-like activity of the fungal cell-free supernatant was assayed using 5 mM of 2,6-dimethoxyphenol (2,6-DMP) (469 nm, Ɛ_469_= 27,500 M^−1^ cm^−1^) in a 100 mM citrate buffer at a pH of 5 in the presence of 50 μL of culture supernatant at 30 °C for 30 s.

### 4.5. Antibacterial Activity Assay

The antibacterial activity of each antibiotic was evaluated before and after treatment using the agar well diffusion method, as described in [55]. Aliquots of 50 µL were collected from the supernatant of each fungal culture containing one antibiotic. In parallel, two controls were used: the first one contained an antibiotic but no fungus, and the second control was the fungal culture without antibiotics. *E. coli* (ATCC 25922) was used as a test strain. All experiments were performed in triplicate. The decrease in the diameter of the inhibition zone was measured every 2 days (from day 4 to day 12). The inhibition efficiencies were calculated by measuring the diameter of growth inhibition against that of the control, as follows:

$$Inhibitionefficiency\left(\%\right)=\frac{\left(D_{0}-D_{t}\right)}{D_{0}}*100$$

where D_0_ and D_t_ denote the diameters of the growth inhibition zone (mm) corresponding to antibiotics injected on day 4 in the culture and the residual antibiotics at culture time t, respectively.

## 5. Conclusions

The main goal of this study was to evaluate the use of the white-rot fungi *C. gallica* to remove tetracycline, chloramphenicol, and sulfanilamide. We revealed significant variations in this strain’s antibiotic degradation efficiency depending on the antibiotic’s structure. *C. gallica* demonstrated complete tetracycline biodegradation and detoxification in both liquid and solid media after 6 days of treatment. However, although the chloramphenicol and sulfanilamide concentrations were partially reduced, the antibiotics were not detoxified, indicating the likely presence of persistent and long-lasting antimicrobial metabolites. An association between laccase production and antibiotic degradation may confirm the involvement of this enzyme, among others, in antibiotic degradation. However, further investigations are needed to determine the specific enzymes involved in these biotransformation reactions to obtain a more complete understanding of tetracycline biodegradation by *C. gallica*. This strain has emerged as a promising candidate for the fungal treatment of antibiotics. Further experiments should be performed to enhance chloramphenicol and sulfanilamide biotransformation by using oxidative enzymes like laccase or the laccase mediator system and to test the ecotoxicity of bio-transformed by-products.

## Figures and Tables

**Table 1:** Physico-chemical characteristics of tetracycline, chloramphenicol, and sulfanilamide.

Antibiotic	Class	Formula	Chemical Structure
Tetracycline	Tetracyclines	C_22_H_24_N_2_O_8_	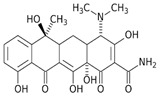
Chloramphenicol	Phenicols	C_11_H_12_Cl_2_N_2_O_5_	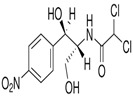
Sulfanilamide	Sulfonamides	C_6_H_8_N_2_O_2_S	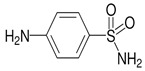

## Data Availability

The original contributions presented in this study are included in the article. Further inquiries can be directed to the corresponding authors.

## Abbreviations

The following abbreviations are used in this manuscript:

2,6-DMP	2,6-dimethoxyphenol
TC	Tetracycline
CHL	Chloramphenicol
SULF	Sulfanilamide
